# Is Laparoscopic Appendectomy Safe When Performed in a Low Volume Setting?

**Published:** 2014-03

**Authors:** Shamir O. Cawich, Sanjib K. Mohanty, Lindberg K. Simpson, Michael J. Ramdass, Vijay Naraynsingh

**Affiliations:** 1 Department of Clinical Surgical Sciences, Faculty of Medicine, University of the West Indies, St. Augustine Campus, Trinidad & Tobago;; 2 Department of Surgery, Health Service Authority, Grand Cayman, BWI;; 3 Department of Surgery, Kingston Public Hospital, Kingston, Jamaica

**Keywords:** Caribbean, Emergency, Appendicitis, Appendectomy, Laparoscopic

## Abstract

**Background:**

Similar to global trends, laparoscopic appendectomy has gained favor across the Caribbean but there is a paucity of published data evaluating its outcomes in the region. This study seeks to document the outcomes of laparoscopic appendectomies performed by community surgeons in a low volume setting in the Caribbean.

**Methods:**

Data were recorded prospectively from all consecutive laparoscopic appendectomies performed from June 1, 2006 to May 30, 2011. Complicated appendicitis was considered present when the appendix was gangrenous, perforated, phlegmonous and/or associated with a peri-appendiceal abscess. Data were analyzed using SPSS version 19.

**Results:**

Appendectomies were performed by one of three surgeons in 167 patients (mean case volume 11 cases per surgeon per year) at mean age of 31.8 ±9.67 years and mean BMI of 29.3 ± 2.59 Kg/m^2^. There was a 14% negative appendectomy rate. Of 143 patients with confirmed appendicitis, 73% were uncomplicated cases and 24% were complicated appendicitis. The mean operating time was 50.1 ±18.4 minutes for uncomplicated cases and 98.8 ±21.6 minutes for complicated appendicitis. The overall morbidity rate was 4.2% (1.8% morbidity in uncomplicated cases and 14.7% for complicated appendicitis. Post-operatively, 69% patients required no supplemental parenteral opioids. After discharge, 7% patients required no oral analgesia and 90% stopped their analgesics within 48 hours.

**Conclusions:**

Laparoscopic appendectomy is a safe operation when performed by community surgeons at low volumes and should be considered as a part of the surgical armamentarium.

## INTRODUCTION

Appendectomy is one of the most common operative procedures performed worldwide (1). Since McBurney (2) described open appendectomy through a right lower quadrant incision in 1891, the operative approach changed minimally until the laparoscopic revolution in the late 1900’s. Semm (3) was the first to perform an appendectomy using the laparoscopic approach in 1983. This approach has since been popularized across the globe, although there is still ongoing debate about its role in the management of acute appendicitis.

Similar to global trends, laparoscopic appendectomy has gained favor across the Caribbean (4) but there is a paucity of published data evaluating its outcomes in the region. This study documents the outcomes of a series of consecutive laparoscopic appendectomies performed by community surgeons in a low volume setting in the Caribbean.

## METHOD

A database of all laparoscopic appendectomies performed by the authors was prospectively maintained from June 1, 2006. The database was accessed to identify all consecutive patients who had appendectomy performed for a pre-operative diagnosis of acute appendicitis between June 1, 2006 and May 30, 2011. Their hospital records were retrieved and the following data collected: patient demographics, body mass index (BMI), pre-operative diagnoses, intra-operative findings, operative details, complications, analgesic requirements and duration of hospitalization. The number of doses of parenteral analgesia administered was used as a proxy for post-operative pain. Data were analyzed using SPSS version 19.

The following complications were recorded: iatrogenic visceral injury, bleeding, superficial wound infection, intra-abdominal collection, stump blowout, urinary retention, respiratory tract infection, pulmonary embolism, fistula formation and port site hernia. Patients were considered to have acute appendicitis when there was inflammation of the vermiform appendix with histologic findings of mucosal ulceration and neutrophil infiltration at the mucosa of the appendix with or without trans-mural extension (5). Complicated appendicitis was considered present when there were intra-operative findings of an appendix that was gangrenous, perforated, phlegmonous and/or associated with a peri-appendiceal abscess (5).

Although there were minor variations in peritoneal access and port placement, the operative techniques were relatively standard. Prophylactic antibiotics were routinely administered at induction. The patients were positioned on the operating table with arms abducted at 90° and the table tilted to Trendelenburg’s position. The monitors were placed at the right side of the bed, with surgeons and assistant standing on the left. Hasson’s technique was used to insert a 10mm visual port at the umbilicus. Two working ports were then inserted under laparoscopic vision at the following locations: 5mm port 1cm medial to the anterior superior iliac spine and a 10mm port 1cm above the pubic symphysis in the midline.

Full laparoscopic exploration of the abdomen was always performed and the small bowel inspected along its entire length to the caecum. The appendix was identified and manipulated with a-traumatic graspers, allowing division of the meso-appendix with electrocautery. The appendix base was ligated with either Endoloop® ligatures (Ethicon Endo-Surgery, USA) or intra-corporeal sutures prior to transection, allowing its removal through the 10mm supra-pubic port. Routine peritoneal lavage with warmed normal saline was carried out in patients with complicated appendicitis and those with iatrogenic contamination. Drains were not routinely used.

Post-operatively, the patients were encouraged to ambulate and have normal diet immediately. Oral analgesia was routinely administered, with parenteral opioids on demand only. The patients were discharged once they tolerated diet and were pain free. We encouraged patients to return to normal activity and exercise immediately after hospital discharge.

## RESULTS

Over the study period there were 167 appendectomies were performed by one of three surgeons through a laparoscopic approach (mean case volume 11 cases per surgeon per year). There were 95 men and 72 women at a mean age of 31.8 ± 9.67 years (range 12-56) and average BMI of 29.3 ± 2.59 Kg/m^2^ (Range 24-38).

In this series, 24 (14%) patients had a normal appendix on histology and another identifiable cause for their acute abdominal pain (tubo-ovarian infections 6; menstrual pain 4; small bowel carcinoids 3; endometriosis 3; adhesive bowel obstruction 2; ureteric calculi 2; urinary tract infections 2; pancreatitis 2). In these patients the appendix was routinely removed and the pathology addressed appropriately.

In the remaining 143 patients, appendicitis was confirmed histologically. Of this, 109 (76%) were uncomplicated cases and 34 (24%) were complicated by abscess formation (10), gangrene (10), perforations (6) and phlegmons (8).

In the group with uncomplicated appendicitis there was a mean operating time of 50.1 ± 18.4 minutes (range 29-120) and 1.8% (2) morbidity. The sole conversion was in this group to repair an iatrogenic injury (serosal tear) to the caecum. In patients with complicated appendicitis, there was a mean operating time of 98.8 ± 21.6 minutes (range 60-150) and 14.7% (5) morbidity.

There were no deaths recorded in this series, but 7 complications occurred, yielding an overall morbidity rate of 4.2%. Two complications (1.5%) occurred in patients with uncomplicated appendicitis and five complications (14.7%) in the group with complicated appendicitis. Table 1 details the individual complications encountered.

**Table 1:** Complications after laparoscopic appendectomy

All Laparoscopic appendectomies (n=167)	7 (4.19%)
Uncomplicated Appendicitis (n = 133)	2 (1.5%)
Superficial wound infection	1 (0.9%)
Serosal tear to caecum	1 (0.9%)
Complicated Appendicitis (n = 34)	5 (14.7%)
Superficial wound infection	1 (2.9%)
Intra-abdominal collection	2 (5.9%)
Respiratory infection	1 (2.9%)
Urinary retention	1 (2.9%)

After laparoscopic appendectomy, 115/167 (69%) patients required no supplemental parenteral opioids after leaving the recovery room. A single dose of parenteral opioid analgesia was administered to 27 (16%) patients and 10 (6%) required >2 doses. After discharge, 12 (7%) patients required no analgesia, 150 (90%) stopped their analgesia within 48 hours and only 17 (10%) required analgesia for >48 hours.

## DISCUSSION

Appendectomy remains the standard treatment for acute appendicitis (1). Open appendectomy through a right iliac fossa incision as popularized by Charles McBurney in 1891 (2) remained the standard of care for a century because of its efficacy and good safety profile (1). But this was challenged in 1983 when Kurt Semm (3) described the performance of appendectomy using the laparoscopic approach.

Over the subsequent thee decades, laparoscopic appendectomy became popular. However, there was continued debate about the role of laparosocopy and its benefits compared to the open approach. Adversaries argued that open appendectomy was effective and the usual benefits of laparoscopy (improved pain profile, shorter hospitalization, reduced respiratory consequences, improved wound healing, rapid return to normal function and cosmesis) were less evident when compared to the good safety profile of an open appendectomy. This was fueled by the results of some prospective randomized trials showing only marginal benefit with laparoscopic appendectomy (7-9) at the expense of greater cost (7-8) and longer operating times (7, 9).

Several meta-analyses of the available prospective randomized trials were commissioned in an attempt to define the role of laparoscopic appendectomy. Most of these meta-analyses documented longer operating times with the laparoscopic approach when done for unselected cases (10-15), with an additional 8 minutes (11) to 18 minutes (15) of theatre time. However, in a prospective randomized trial evaluating 244 patients with complicated appendicitis, Yau *et al*. (16) demonstrated that laparoscopy significantly reduced the operating time by 15 minutes when compared to the open approach. There were no data on open appendectomy from the Caribbean region with which we could compare operating time. However, the time to complete laparoscopic appendectomy (50.1 ± 18.4 minutes) in uncomplicated cases was comparable to existing reports from high volume centres (6-9, 16, 17), that ranged from 36 minutes (17) to 80 minutes (9).

Despite longer operating times, most recent meta-analyses have demonstrated a definite advantage with the laparoscopic approach over open appendectomy (1, 10-15, 18-25), with significant reductions in wound infections (1, 10, 12, 14, 15, 18, 19) and overall morbidity (13-15). The overall morbidity (4.2%) and overall superficial wound infection rates (1.2%) in this series were relatively low and they were comparable to figures published in the large volume meta-analyses (10-15, 18, 19).

Most authorities agree that there is a significant reduction in superficial wound infections with the laparoscopic approach (1, 10, 12, 14, 15, 18, 19), ranging from 0.6% (16) to 3.8% (1) of cases. However, there is still conflicting data on the incidence of deep intra-abdominal collections, with some meta-analyses suggesting a significant rise with laparoscopy (1, 10, 12) and many showing similar incidence regardless of the approach (11, 15, 16, 19). Again, there were no reports on the outcomes of open appendectomy from the Caribbean, but the incidence of intra-abdominal collections after laparoscopic appendectomy for complicated appendicitis (5.9%) was comparable to that reported from high volume centres, ranging from 5.7% (16) to 19.1% (20) in complicated cases.

Most recent meta-analyses also demonstrate a definite advantage with the laparoscopic approach over open appendectomy with significant reductions in pain (1, 11-14), hospitalization (1, 10-15, 21) and time to return to full activity (1, 11-14). Laparoscopic appendectomy in our setting had good pain profiles, with 85% of patients requiring ≤1 dose of parenteral analgesia post-operatively and 90% discontinuing their oral analgesia within 48 hours of operation.

There is also conflicting data on cost, with some meta-analyses showing an increase in operational costs with laparoscopy (12) and others reporting no difference (13, 14). The nature of this study did not allow us to analyze cost associated with laparoscopic appendectomy and there were no existing data on costs associated with open appendectomy in the Caribbean either.

Regardless of increased cost, operating time or intra-abdominal infections, the overall thrust of recent high quality data is in support of routine laparoscopic appendectomy, especially for young females, obese patients and those with complicated appendicitis. Interestingly, the latest Cochrane database suggested that this should also be the standard of care for “employed patients” who need to return to normal activity rapidly (12). As testimony to the support for routine laparoscopic appendectomy, the debate has now migrated away from the benefit of laparoscopic appendectomy, now focusing on comparisons between conventional three-port laparoscopic appendectomy versus single incision laparoscopic appendectomy (22-24) and needlescopic appendectomy (25). There has been no report of these advanced modalities being employed for appendectomy in the Caribbean to date.

Despite the well-documented challenges to the incorporation of minimally invasive approaches to surgical diseases in the Caribbean (26), laparoscopic appendectomy continues to be employed by community surgeons with good outcomes. The mean case volumes were low at only 11 laparoscopic appendectomies per surgeon per year. We expect the case volumes to increase and the outcomes to improve with the recent initiatives aimed at promoting minimally invasive surgery across the region: 1) the establishment of centres of excellence across the Caribbean; 2) the inception of the Caribbean Society of Endo-Laparoscopic Surgeons - a professional association specifically geared toward laparoscopic surgery; and 3) numerous collaborative training initiatives between the Caribbean College of Surgeons and the Royal College of Surgeons.

## CONCLUSIONS

Laparoscopic appendectomy is a feasible and safe operation when performed by community surgeons at low volumes in this setting.

## STUDY LIMITATIONS

Although this is the first report of laparoscopic appendectomy from the region, it has relatively small patient numbers. Nevertheless, we believe it is important to analyze these outcomes to have a realistic appreciation for the outcomes of repair in the hands of community surgeons who are performing these operations infrequently.
